# Benefits and harms of ADHD interventions: umbrella review and platform for shared decision making

**DOI:** 10.1136/bmj-2025-085875

**Published:** 2025-11-27

**Authors:** Corentin J Gosling, Miguel Garcia-Argibay, Michele De Prisco, Gonzalo Arrondo, Anaël Ayrolles, Stéphanie Antoun, Serge Caparos, Ana Catalán, Pierre Ellul, Maja Dobrosavljevic, Luis C Farhat, Giovanna Fico, Luis Eudave, Annabeth P Groenman, Mikkel Højlund, Lucie Jurek, Mikail Nourredine, Vincenzo Oliva, Valeria Parlatini, Constantina Psyllou, Gonzalo Salazar-de-Pablo, Anneka Tomlinson, Samuel J Westwood, Andrea Cipriani, Christoph U Correll, Dong Keon Yon, Henrik Larsson, Edoardo G Ostinelli, Jae Il Shin, Paolo Fusar-Poli, John P A Ioannidis, Joaquim Radua, Marco Solmi, Richard Delorme, Samuele Cortese

**Affiliations:** 1 Laboratoire DysCo, Université Paris Nanterre, 92000 Nanterre, France; 2 Department of Child and Adolescent Psychiatry, Institut Robert Debré du Cerveau de l’enfant Robert Debré Hospital, APHP, Paris, France; 3 Developmental EPI (Evidence synthesis, Prediction, Implementation) Laboratory, Centre for Innovation in Mental Health (CIMH), School of Psychology, Faculty of Environmental and Life Sciences, University of Southampton, UK; 4 Hampshire and Isle of Wight Healthcare NHS Foundation Trust, Southampton, UK; 5 School of Medical Sciences, Faculty of Medicine and Health, Örebro University, Örebro, Sweden; 6 Department of Medical Epidemiology and Biostatistics, Karolinska Institutet, Stockholm, Sweden; 7 Bipolar and Depressive Disorders Unit, Hospital Clínic de Barcelona, Imaging of Mood and Anxiety-Related Disorders (IMARD) Group, Institut d'Investigacions Biomèdiques August Pi i Sunyer (IDIBAPS), CIBERSAM, Barcelona, Spain; 8 Mind-Brain Group, Institute for Culture and Society (ICS), University of Navarra, Pamplona, Spain; 9 Human Genetics and Cognitive Functions, Institut Pasteur, Paris, France; 10 Université Paris 8, Laboratoire DysCo, Saint-Denis, France; 11 Psychiatry Department, Biocruces Bizkaia Health Research Institute, OSI Bilbao-Basurto, Facultad de Medicina y Odontología, University of the Basque Country UPV/EHU, Centro de Investigación en Red de Salud Mental (CIBERSAM), Instituto de Salud Carlos III, Barakaldo, Bizkaia, Spain; 12 Early Psychosis: Interventions and Clinical-detection (EPIC) Lab, Department of Psychosis Studies, King’s College London, UK; 13 Department of Brain and Behavioural Sciences, University of Pavia, Italy; 14 Université Paris-Cité, Paris, France; 15 3I Brain- Immune system Brain interactions in physiology, infections or inflammation, INSERM U1141 NeuroDiderot, Paris, France; 16 Department of Psychiatry, Faculdade de Medicina FMUSP, Universidade de Sao Paulo, Sao Paulo, Brazil; 17 Bipolar and Depressive Disorders Unit, Hospital Clinic de Barcelona, Barcelona, Spain; 18 Institut d’Investigacions Biomèdiques August Pi i Sunyer (IDIBAPS), Barcelona, Spain; 19 Departament de Medicina, Institut de Neurociències (UBNeuro), Universitat de Barcelona (UB), Spain; 20 School of Education and Psychology, University of Navarra, Pamplona, Spain; 21 Research Institute of Child Development and Education, University of Amsterdam, Amsterdam, Netherlands; 22 Department of Child and Adolescent Psychiatry, University Medical Center Groningen, University of Groningen, Groningen, Netherlands; 23 Accare Child Study Center, Groningen, Netherlands; 24 Unit of Mental Health Research, Southwest Denmark, Department of Regional Health Services, University of Southern Denmark, Odense, Denmark; 25 Clinical Pharmacology, Pharmacy, and Environmental Medicine, Department of Public Health, University of Southern Denmark, Odense, Denmark; 26 Child and Adolescent Mental Health Centre, Mental Health Services Capital Region of Denmark, Copenhagen, Denmark; 27 Pôle de Psychiatrie de l’Enfant et de l’Adolescent, Centre Hospitalier Le Vinatier, Bron, France; 28 Service de Biostatistique-Bioinformatique, Hospices Civils de Lyon, Lyon, France; 29 Centro de Investigación Biomédica en Red de Salud Mental (CIBERSAM), Instituto de Salud Carlos III, Madrid, Spain; 30 Department of Child and Adolescent Psychiatry, Institute of Psychiatry, Psychology and Neuroscience, King’s College London, London, UK; 31 Department of Forensic and Neurodevelopmental Sciences, Institute of Psychiatry, Psychology and Neuroscience, King’s College London, London, UK; 32 School of Psychology, University of Southampton, Southampton, UK; 33 Child and Adolescent Mental Health Services (CAMHS), South London and Maudsley NHS Foundation Trust, London, UK; 34 Department of Child and Adolescent Psychiatry, Institute of Psychiatry and Mental Health, Hospital General Universitario Gregorio Marañón School of Medicine, Universidad Complutense, IiSGM, CIBERSAM, Madrid, Spain; 35 Department of Psychiatry, Warneford Hospital, University of Oxford, Oxford, UK; 36 Oxford Precision Psychiatry Lab, National Institute for Health and Care Research Oxford Health Biomedical Research Centre, Oxford, UK; 37 Department of Psychology, Institute of Psychiatry, Psychology, and Neuroscience, King’s College London, London, UK; 38 NIHR Oxford Health Clinical Research Facility, Oxford Health NHS Foundation Trust, Warneford Hospital, Oxford, UK; 39 Department of Child and Adolescent Psychiatry, Charité Universitätsmedizin, Berlin, Germany; 40 Department of Psychiatry, Zucker Hillside Hospital, Glen Oaks, NY, USA; 41 Department of Psychiatry and Molecular Medicine, Donald and Barbara Zucker School of Medicine at Hofstra/Northwell, Hempstead, NY, USA; 42 Center for Psychiatric Neuroscience, Feinstein Institute for Medical Research, Manhasset, NY, USA; 43 German Center for Mental Health (DZPG), partner site Berlin, Berlin, Germany; 44 German Center for Child and Adolescent Health (DZKJ), partner site Berlin, Berlin, Germany; 45 Department of Pediatrics, Kyung Hee University Medical Center, Kyung Hee University College of Medicine, Seoul, South Korea; 46 Severance Underwood Meta-Research Center, Institute of Convergence Science, Yonsei University, Seoul, South Korea; 47 Department of Pediatrics, Yonsei University College of Medicine, Seoul, South Korea; 48 Outreach and Support in South-London (OASIS) service, South London and Maudsley (SLaM) NHS Foundation Trust, UK; 49 Department of Psychiatry and Psychotherapy, University Hospital, Ludwig-Maximilian-University (LMU), Munich, Germany; 50 Departments of Medicine, of Epidemiology and Population Health, and of Biomedical Data Science, and Meta-Research Innovation Center at Stanford (METRICS), Stanford University, Stanford, CA, USA; 51 Ottawa Hospital Research Institute, Ottawa, Ontario, Canada; 52 Psychiatry Department, University of Ottawa, Ottawa, Ontario, Canada; 53 The Ottawa Hospital, Ottawa, Ontario, Canada; 54 Clinical and Experimental Sciences (CNS and Psychiatry), Faculty of Medicine, University of Southampton, Southampton, UK; 55 Hassenfeld Children’s Hospital at NYU Langone, New York University Child Study Center, New York City, NY, USA; 56 DiMePRe-J Department of Precision and Regenerative Medicine, Jonic Area, University of Bari “Aldo Moro,” Bari, Italy

## Abstract

**Objectives:**

To assess the effects of and related evidence certainty of interventions for attention deficit/hyperactivity disorder (ADHD) across an individual’s lifespan, and to develop a continuously updated web platform for people with lived experience of ADHD as a method to disseminate living evidence synthesis for shared decision making.

**Design:**

Umbrella review and platform for shared decision making.

**Data sources:**

Six databases from inception to 19 January 2025. Study authors were contacted for additional information when necessary.

**Eligibility criteria for selecting studies:**

Systematic reviews that used meta-analyses of randomised controlled trials were eligible if they compared a drug or non-drug intervention with a passive control in individuals with a diagnosis of ADHD. Primary outcomes were severity of ADHD symptoms, analysed by rater type (clinician-rated, parent-rated, teacher-rated, or self-rated) and time point (short term (12 weeks, or study endpoint), medium term (26 weeks), and long term (52 weeks)),acceptability (participants dropping out for any reason), and tolerability (participants dropping out owing to any side effects). Secondary outcomes included daily functioning, quality of life, comorbid symptoms, and key side effects (decreased sleep and appetite).

**Data synthesis:**

Eligible meta-analyses were re-estimated with a standardised statistical approach. Methodological quality was assessed using AMSTAR-2. Evidence certainty was evaluated using an algorithmic version of the GRADE framework, adapted for drug and non-drug interventions.

**Results:**

115 of 414 full text articles were deemed eligible and 299 were excluded; the eligible articles comprised 221 unique combinations of participants, interventions, comparators, and outcomes. For each combination, the most recent and methodologically robust meta-analysis was selected for re-estimation, which gave 221 re-estimated meta-analyses in total, derived from 47 meta-analytic reports. In the short term, alpha-2 agonists, amphetamines, atomoxetine, methylphenidate, and viloxazine showed medium to large effect sizes in reducing the severity of ADHD symptoms in children and adolescents, with moderate to high certainty evidence. Methylphenidate showed consistent benefits across raters (standardised mean difference >0.75, 95% confidence interval (CI) 0.56 to 1.03; moderate or high certainty evidence). These interventions showed lower tolerability than the placebo, but this effect was not significant for methylphenidate and atomoxetine. In adults, atomoxetine, cognitive behavioural therapy, methylphenidate (and, when restricting analyses to high quality trials, amphetamines) showed at least moderate certainty evidence of efficacy on ADHD symptoms, with medium effect sizes. Methylphenidate, amphetamines, and atomoxetine had worse tolerability than placebo (methylphenidate, risk ratio 0.50, 95% CI 0.36 to 0.69; amphetamines, 0.40, 0.22 to 0.72; atomoxetine, 0.45, 0.35 to 0.58). Some non-drug interventions (acupuncture and cognitive behavioural therapy in children and adolescents, and mindfulness in adults) showed large effect sizes for ADHD symptoms, but with low certainty evidence. No high certainty, long term evidence was found for any intervention. An online platform showing effects and evidence certainty of each intervention across age groups, time points, and outcomes (https://ebiadhd-database.org/) was developed.

**Conclusions:**

This review provides updated evidence to inform patients, practitioners, and guideline developers how best to manage ADHD symptoms. The online platform should facilitate the implementation of shared decision making in daily practice.

**Trial registration:**

Open Science Framework https://osf.io/ugqy6/.

## Introduction

Attention deficit/hyperactivity disorder (ADHD) is characterised by persistent and impairing difficulties with attention or hyperactivity-impulsivity, or both,^1^ ^2^ and is estimated to affect 5% of children, with impairing symptoms continuing into adulthood in up to 70% of cases.^3^ ADHD frequently coincides with other disorders, including mood disorders, anxiety disorders, and substance use disorders, as well as impairments, such as emotional dysregulation and executive dysfunction.^2^ ADHD is associated with negative outcomes in patients, including lower academic achievement, impaired interpersonal functioning, increased risk of physical injuries or road traffic incidents, and reduced quality of life.^4^ ^5^ The disorder is associated with substantial economic burden, with annual estimates for excess societal costs in the US of $19.4bn (£14.5bn; €16.7) for children, $13.8bn for adolescents,^6^ and $122.8bn for adults.^7^

Available interventions proposed to manage ADHD symptoms include drugs such as stimulants (methylphenidate and amphetamines) and non-stimulants (eg, atomoxetine, bupropion, clonidine, guanfacine, and viloxazine),^8^ as well as non-drug strategies (eg, behavioural therapies, dietary interventions, and neurofeedback).^9^ Current guidelines generally recommend drugs (typically with stimulants as the preferred treatment and non-stimulants as second line treatment) alongside behavioural or cognitive behavioural interventions.^10^

Numerous meta-analyses of randomised controlled trials have evaluated ADHD interventions, yet the practical utility of these meta-analyses for busy clinicians remains limited. Firstly, available meta-analyses often focus on selected interventions or outcomes, and fail to address questions from practitioners and other stakeholders. Secondly, existing meta-analyses frequently show conflicting results about the effects of the same intervention—for example, neurofeedback or cognitive training.^11^ ^12^ Furthermore, many meta-analyses fail to rate the quality of the evidence, slowing the implementation of evidence based management of ADHD.^13^ In addition, the dissemination of meta-analytical results is typically limited to publication in academic journals. This lack of accessible information for people with lived experience of ADHD has led them to explicitly request an online resource that reports high quality scientific evidence, particularly as current online resources usually lack details on non-drug interventions and management of comorbidities.^14^ ^15^

As a result, a comprehensive review was needed to evaluate the effects of ADHD treatments across outcomes and age groups, associated with the quality of evidence for such interventions. In addition, this information would need to be effectively disseminated. In line with the recently proposed umbrella review, evaluation, analysis, and communication hub (U-REACH) framework,^16^ we conducted an umbrella review on interventions for children, adolescents, and adults with ADHD. Available treatments and outcomes (eg, ADHD symptoms, quality of life) were evaluated. To improve the accessibility and clinical translation of our umbrella review, we developed an open access online platform showing our results in a user friendly format for clinicians, service users, guideline developers, and other stakeholders.

## Methods

We conducted this study following the U-REACH framework.^16^ The methods of this umbrella review were pre-registered (https://osf.io/ugqy6/), and they are reported according to relevant guidelines.^16^ ^17^ ^18^ Details can be found in supplementary files S1 and S2, along with any protocol deviations.

### Umbrella review methodology

Screening, data extraction, and methodological quality assessment of the meta-analyses were performed by two independent investigators and verified by CJG and MGA. Senior authors resolved any disagreements (MS, RD, and SCo). 

### Search strategy and eligibility criteria

Two information specialists searched PubMed, Embase, Emcare, PsycInfo, Web of Science, and Cochrane Library from inception to 19 January 2025 for systematic reviews that meta-analysed data from randomised controlled trials in individuals with ADHD. No restrictions were applied by language or document type (eg, conference proceedings).

A review was considered systematic if the authors identified it as such, searches of at least two electronic databases were included, and explicit inclusion and exclusion criteria were used. Pairwise, network, and individual participant data meta-analyses were eligible for inclusion (supplementary file S3). In addition, we manually screened the references of included reviews and consulted with experts to identify any further eligible reviews. In the case of multiple meta-analyses of the same combination of participants, interventions, comparators, and outcomes, we selected the most recent and highest quality meta-analysis for re-estimation (supplementary methods S4).

Participants in randomised controlled trials were required to have a diagnosis of ADHD based on the *Diagnostic and Statistical Manual of Mental Disorders*, versions III to 5-TR or ICD (international classification of diseases) ninth to 11th revisions (supplementary file S5). No age restrictions applied, but results were presented separately for three age groups: preschoolers, children and adolescents, and adults. We included any available interventions, provided they were compared with passive conditions. Details on our exact inclusion criteria are available in supplementary file S5, and a complete description of the interventions is available at https://ebiadhd-database.org/interventions/.

Primary outcomes included the severity of combined ADHD symptoms (inattention-hyperactivity-impulsivity) analysed separately by the person providing the symptom assessment (hereafter referred to as the rater—parents/caregivers, clinicians, teachers, or self-report—whenever possible; when the rater could not be reliably identified, the category mixed was used), acceptability (participants dropping out for any reason), and tolerability (participants dropping out owing to any side effects).

Secondary outcomes included the reported level of functioning (eg, academic or work productivity), symptom severity of comorbid disorders or conditions, quality of life, suicidal ideation or behaviour, and the two selected side effects of decreased appetite and sleep problems.

The results of all outcomes are presented separately according to the timing of the measure: short term (ie, usually close to 12 weeks after the start of the study, at the first study endpoint) and medium to long term (ie, at the time point closest to 26 weeks or 52 weeks, usually at follow-up, as with earlier reviews^19^; supplementary methods S5). When the timing of the measure could not be reliably identified, we assumed it occurred at the end of the 12 weeks study endpoint.

### Data extraction and checking

We extracted information on the characteristics of the trial design, participants, interventions, and intervention effects of each randomised controlled trial from meta-analytic reports. When participants’ age was not given in the report, we searched for this information in the relevant randomised controlled trial. When the estimated effect size from a randomised controlled trial was found to be implausible (eg, a standardised mean difference ≥3), we tried to reproduce the calculations made by the authors of the meta-analysis using the metaConvert tool,^20^ and excluded meta-analytical studies containing clear inaccuracies or errors.

### Assessment of the methodological quality of primary studies and meta-analyses

In accordance with current guidelines, and similarly to other umbrella reviews,^16^ ^17^ ^18^ ^21^ we assessed the methodological quality of the primary randomised controlled trials by extracting relevant information from the meta-analytic reports. The methodological quality of the meta-analyses was evaluated using AMSTAR-2.^22^

### Assessment of the certainty of evidence

The certainty of evidence for the effects of each intervention was assessed using a recent algorithmic adaptation of GRADE (grading of recommendations, assessment, development and evaluation).^23^ ^24^ ^25^ This approach allows for classification of the certainty of evidence into four ordinal classes: high, moderate, low, and very low. Certain methodological biases are inherently difficult to address in randomised controlled trials of non-drug interventions, such as the blinding of participants and investigators. We defined a study as having a high risk of bias if the risk of bias was rated as high for at least one of three key criteria (applicable to both drug and non-drug randomised controlled trials): randomisation process (eg, concealment of allocation to either intervention or control group), outcome measurement (eg, lack of outcome assessor blinding), and selection of the reported outcome (eg, cherry picking of the outcome measure) (supplementary file S6).

### Data analysis

Because meta-analyses sometimes used different data analysis strategies (such as fixed effects versus random effects models, or Der-Simonian Laird versus restricted maximum likelihood estimator), we re-estimated each meta-analysis using the same statistical approach in our primary analysis. R software (version 4.3.1) and the “metaumbrella” package were used for analysis.^25^ We used standardised mean difference for continuous outcomes, and risk ratio for dichotomous outcomes. In the context of meta-analyses of mean difference and odds ratio, we conducted a harmonisation of effect size metrics through the conversion of individual randomised controlled trial effect sizes into standardised mean difference and risk ratio, respectively. This harmonisation was performed using the metaConvert tool before conducting calculations.^25^ For meta-analyses of odds ratio and risk ratio, we performed a re-estimation of the effect size from the contingency table when available. This approach was necessary because the analytical strategies used in meta-analytical reports to deal with the situation of no events in one or two groups often differed. To manage these zero-events situations, a continuity correction was performed whereby a +0.5 correction was applied to all cells of the contingency table. Irrespective of the metric used (risk ratio or standardised mean difference), the direction of the effect was reversed when necessary, such that a risk ratio <1 or a standardised mean difference >0 systematically reflected an improvement: reduction in symptoms, improvement in everyday functioning, or greater safety. The change in direction was systematically completed after effect size and variance calculations. We used random effects models with restricted maximum likelihood for standardised mean difference and Paul-Mandel method for risk ratio. Heterogeneity was primarily assessed using prediction intervals, and we evaluated small study using Egger’s test,^26^ excess significance bias,^27^ and proportion of participants in studies with high risk of selective reporting. A corrected covered area analysis was also conducted to determine the degree of overlap in primary studies included across the meta-analyses.^28^

A sensitivity analysis was performed on randomised controlled trials assessed as having a low risk of bias. We did this to ensure that our grading criteria—which downgraded evidence quality based on the percentage of participants in studies at high risk of bias—did not unduly penalise meta-analyses that included high and low quality randomised controlled trials (supplementary file S7).

The prespecified secondary analysis that aimed to re-estimate or use existing network meta-analyses with head to head comparisons was not completed. When we considered the randomised controlled trials that met our stringent inclusion criteria, many network meta-analyses showed star shaped network structures (ie, trials predominantly connected by a common comparator, typically the placebo). Therefore, we did not report the results of this secondary analysis.

### Platform development and sustainability plans

To disseminate our results in a user friendly manner, we developed the evidence based interventions for ADHD (EBI-ADHD) platform (https://ebiadhd-database.org). The platform is divided into three sections: intervention, preference, and database pages. The intervention section gives an overview of each identified intervention, and details the population in which it has been studied as well as its efficacy and safety across all assessed outcomes. The preference section provides a complete map of available evidence for the efficacy and safety of all interventions, grouped by age group and follow-up duration. This resource helps clinicians, people with lived experience of ADHD, and families of those affected by ADHD to make informed decisions about their symptom management. Finally, the database section allows researchers and guideline developers to visualise the entire dataset of results and identify gaps in knowledge.

Our review and its associated platform have been developed as a method of living evidence synthesis. We are committed to updating the data and web resource annually, adhering to the methodology described in this paper. These updates are scheduled to continue throughout the current funding period of this project, which concludes at the end of 2028. To ensure the long term sustainability and utility of this resource, we plan to actively pursue additional funding opportunities. Furthermore, we will explore the use of artificial intelligence in potentially enhancing the efficiency and scope of future updates, aiming to maintain this living umbrella review as a continuously relevant evidence base for ADHD interventions.

### People with lived experience and public involvement

Based on the primary results of our review, we developed a preliminary version of the online platform inspired by our team’s earlier work.^29^ We also sought feedback from key stakeholders, including representatives from ADHD Europe, the largest advocacy organisation for people with ADHD in Europe, and clinicians within our research network. We emailed people with lived experience of ADHD and clinical practitioners, and subsequently shared Google documents with them over a period of three months, allowing them to contribute feedback at their convenience and minimising any burden on their time. Their feedback focused specifically on the usability, clarity, and organisation of the online platform. This input led to substantial improvements in the website’s navigation system and the data visualisation (plotting) system, ensuring that our findings were presented in a more user friendly and understandable format.

We plan to continue engaging with key stakeholders for future updates of the umbrella review and platform, ensuring that the information remains relevant and accessible and addresses the changing needs of service users, their families, and clinical practitioners.

## Results 

**Fig 1:**
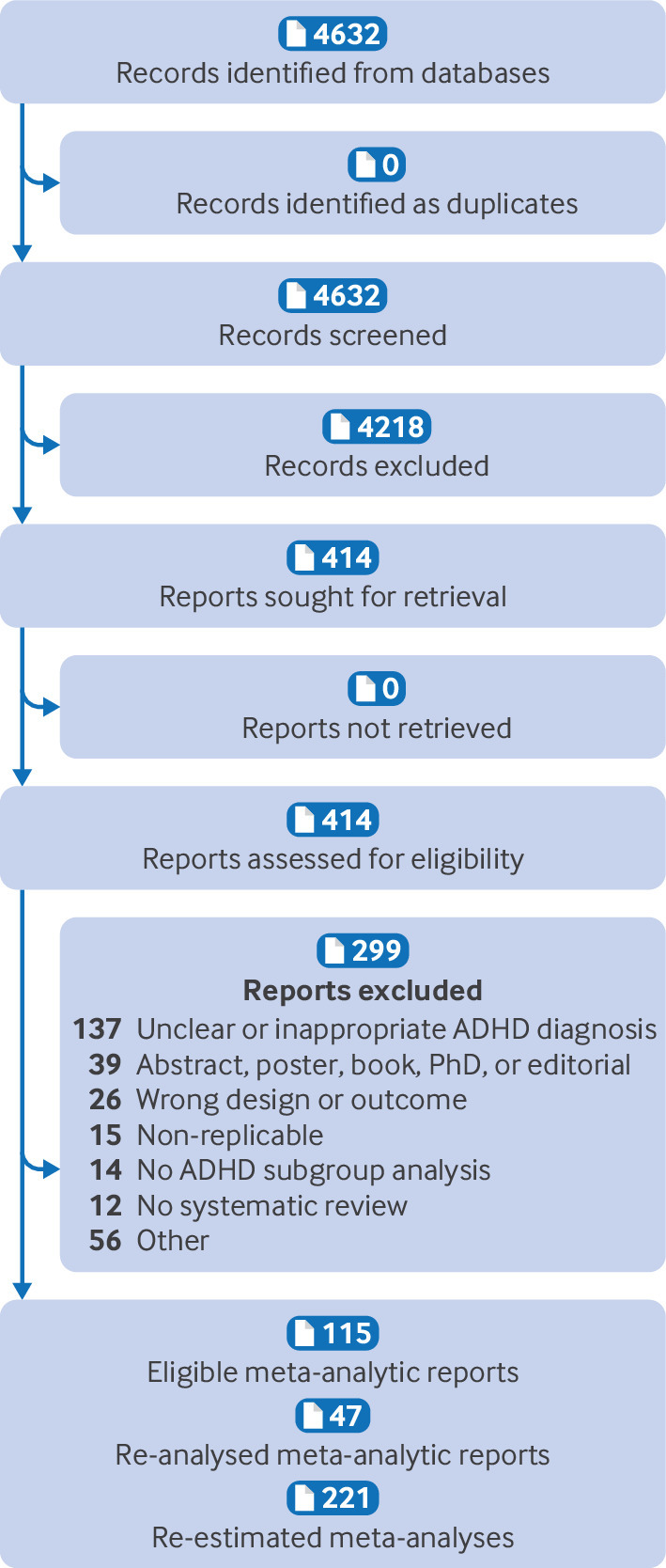
Flow diagram

After screening 4632 references, we assessed 414 full text articles, of which 115 meta-analytical reports were deemed eligible for inclusion (supplementary file S8). These 115 reports described 221 unique combinations of participants, interventions, comparators, and outcomes, exploring the effect of 31 interventions on 24 outcomes. The most recent and methodologically robust meta-analysis for each combination of participants, interventions, comparators, and outcomes was selected for re-estimation, giving a final sample of 221 re-estimated meta-analyses of each unique combination derived from 47 distinct meta-analytic reports. Figure 1 shows the flow of articles (n=115) through the review, with reasons for exclusion of 299 articles (see supplementary file S9 for details).

### Characteristics of meta-analyses 

The 221 re-estimated meta-analyses of each combination of participants, interventions, comparators, and outcomes explored the effects of at least one of 31 interventions, on at least one of 24 outcomes. Using AMSTAR-2, 59% (n=131) of these 221 meta-analyses were deemed to be of high methodological quality, 7% (n=15) of moderate quality, and 34% (n=75) of low or critically low quality. Supplementary file S10 provides further details on AMSTAR-2 scoring and the corrected covered area analysis used to assess overlap in primary studies.

### Primary outcomes

#### ADHD symptoms

**Fig 2:**
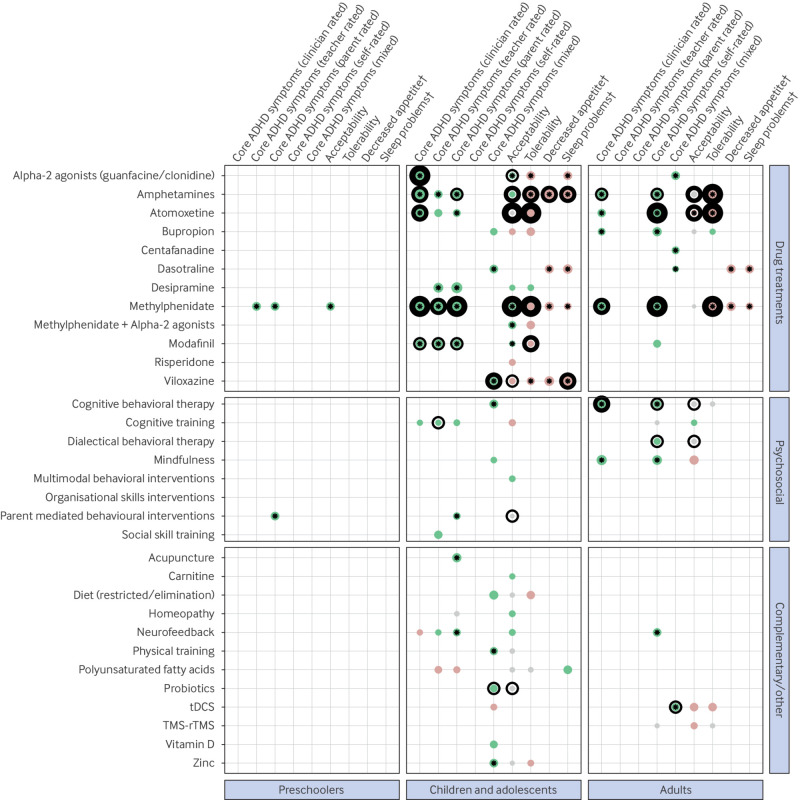
Scatter plot showing the direction of the pooled effect sizes for primary outcomes and key side effects for each combination of participants, interventions, comparators, and outcomes. Grey represents an absence of clinically relevant effect (−0.20 <standardised mean difference <0.20, 0.80 <risk ratio <1.25), green represents a positive effect, and red represents a negative effect. The wider the dots, the larger the pooled effect size. *P<0.05 represents statistical significance. Evidence certainty rating: no surrounding ring=very low certainty, thin surrounding ring=low certainty, bold surrounding ring=moderate certainty, large bold surrounding ring=high certainty. †Side effect. ADHD=attention deficit/hyperactivity disorder. tDCS=transcranial direct current stimulation. TMS-rTMS= (repetitive) transcranial magnetic stimulation

In the short term, in children and adolescents, five drugs showed statistically significant improvements in ADHD symptoms with at least moderate certainty evidence (fig 2; supplementary file S11). Methylphenidate showed consistent benefits across raters (standardised mean difference >0.75, 95% confidence interval (CI) 0.56 to 1.03; moderate or high certainty evidence). For amphetamines, clinicians’ ratings showed large effects (1.02, 0.67 to 1.38; evidence certainty moderate) but parents’ and teachers’ ratings showed smaller effects (standardised mean difference <0.60 for both groups; low or very low certainty evidence). Among non-stimulants, alpha-2 agonists (0.64, 0.48 to 0.79; high certainty evidence) and atomoxetine (0.53, 0.41 to 0.64; moderate certainty evidence) showed medium to large effects according to clinicians’ ratings. Viloxazine showed small to medium improvements for mixed raters (0.38, 0.26 to 0.49; moderate certainty evidence). Desipramine showed large effect sizes on ADHD symptoms, based on parents’ and/or teachers’ ratings (standardised mean difference >0.65, P<0.05), although with very low certainty evidence. We found the same pattern of results for methylphenidate in preschoolers. None of the non-drug interventions reached high or moderate certainty evidence, despite acupuncture and cognitive behavioural therapy showing statistically significant, apparently large effect sizes.

In adults, only two drugs and cognitive behavioural therapy showed at least medium efficacy on ADHD symptoms, with moderate to high certainty evidence (self-reported: methylphenidate, standardised mean difference 0.34, 0.26 to 0.42, evidence certainty high; atomoxetine, 0.37, 0.26 to 0.47, evidence certainty high; clinician rated: methylphenidate, 0.50, 0.39 to 0.61, evidence certainty moderate; cognitive behavioural therapy, 0.53, 0.29 to 0.77, evidence certainty moderate). Some other interventions (alpha-2 agonists, bupropion, and mindfulness) had apparently large effects (standardised mean difference >0.65, P<0.05), but with low certainty or very low certainty evidence.

At medium/long term follow up, regardless of intervention type or age group, we found no high or moderate certainty evidence (supplementary file S13). Only mindfulness in adults showed apparently large effects on self-reported ADHD symptoms at medium term follow up, but with very low certainty evidence.

#### Acceptability, tolerability, and side effects

In children and adolescents, amphetamines showed worse tolerability than placebo (risk ratio 0.46, 95% CI 0.22 to 0.96; moderate certainty evidence), whereas atomoxetine (high certainty evidence), methylphenidate (high certainty evidence), and modafinil (moderate certainty evidence) were not significantly different from placebo, despite all pooled risk ratio values ranging from 0.54 to 0.79 (supplementary file S11). Methylphenidate showed significantly better acceptability than placebo (1.58, 1.35 to 1.85; high certainty evidence), whereas atomoxetine and amphetamines showed no significant difference (risk ratios 1.00 to 1.23 for both drugs) with high and moderate certainty evidence for atomoxetine and amphetamines, respectively. Viloxazine had moderate certainty evidence for side effects on sleep (risk ratio 0.27, 0.16 to 0.46).

In adults, drug interventions with at least moderate certainty evidence of favourable effects on ADHD symptoms had high certainty evidence of worse tolerability than placebo (methylphenidate, risk ratio 0.50, 0.36 to 0.69; atomoxetine, 0.45, 0.35 to 0.58). Acceptability and tolerability were rarely assessed for non-drug interventions.

#### Secondary outcomes

**Fig 3:**
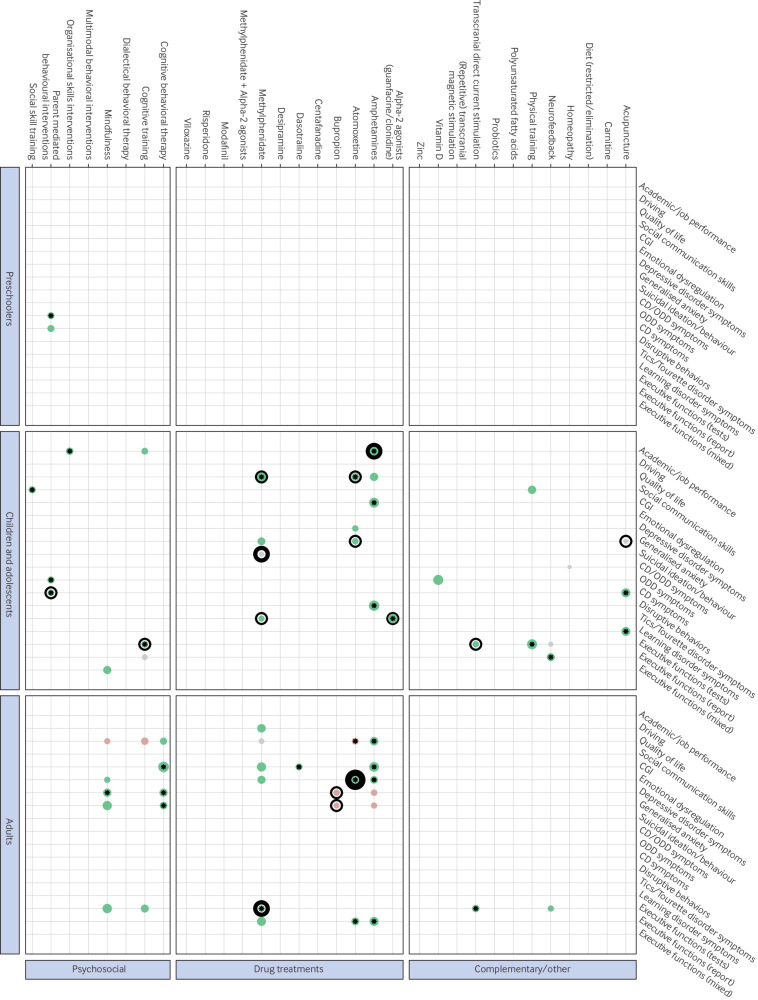
Scatter plot showing the direction of the pooled effect sizes for secondary outcomes for each combination of participants, interventions, comparators, and outcomes. Grey represents an absence of clinically relevant effect (−0.20 <standardised mean difference <0.20, 0.80 <risk ratio <1.25), green represents a positive effect, and red represents a negative effect. The wider the dots, the larger the pooled effect size. *P<0.05 represents statistical significance. Evidence certainty rating: no surrounding ring=very low certainty, thin surrounding ring=low certainty, bold surrounding ring=moderate certainty, large bold surrounding ring=high certainty. CGI=Clinical Global Impressions. tDCS=transcranial direct current stimulation. TMS-rTMS= (repetitive) transcranial magnetic stimulation. CD/ODD=conduct disorder (CD)/oppositional defiant disorder (ODD)

In the short term, only a few interventions were supported by moderate or high certainty evidence (fig 3). In children and adolescents, amphetamines showed medium improvements in academic performance (standardised mean difference 0.55, 95% CI 0.37 to 0.73; moderate certainty evidence) and atomoxetine showed small to medium improvements on quality of life (0.33, 0.19 to 0.48; moderate certainty evidence). Methylphenidate was not significantly different from placebo for suicidal ideation or behaviour in children and adolescents (risk ratio 1.10, 95% CI 0.24 to 4.96, moderate certainty evidence). In adults, atomoxetine showed small improvements on emotional dysregulation (standardised mean difference 0.24, 95% CI 0.14 to 0.34; high certainty evidence) and methylphenidate showed small improvements on executive functions (0.15, 0.03 to 0.28; moderate certainty evidence). Many other interventions showed apparently large, significant effect sizes on some secondary outcomes, but with low certainty evidence (acupuncture, alpha-2 agonists, amphetamines, and physical training in children; amphetamines, cognitive behavioural therapy, and mindfulness in adults; supplementary file S12).

At medium/long term follow up, we found no high certainty or moderate certainty evidence on any of the secondary outcomes. Only mindfulness in adults had apparently large effects (on executive functioning), but with very low certainty evidence (supplementary file S13).

### Sensitivity analysis

Analysis restricted to studies with low risk of bias showed only small differences compared with our main analysis. The main difference we observed was for amphetamines: the favourable effects on ADHD symptoms (in both children/adolescents and adults) and unfavourable effects on sleep and appetite (in children/adolescents) were of similar magnitude but were upgraded from very low or low certainty evidence to moderate or high certainty evidence (supplementary file S14). 

### Web platform

The EBI-ADHD open access web platform (https://ebiadhd-database.org/) gives the results from the 221 meta-analyses used in our primary analysis in an interactive format, with detailed descriptions of each intervention. Users can filter population characteristics, view effect sizes with confidence intervals, and access evidence certainty ratings. All findings are accompanied by plain language summaries.

## Discussion

### Principal findings 

We conducted a comprehensive quantitative synthesis of randomised evidence on drug and non-drug interventions for the management of ADHD symptoms, based on 221 meta-analyses of randomised controlled trials. For drug treatments, we found moderate to high certainty evidence supporting the short term efficacy of alpha-2 agonists, amphetamines, atomoxetine, methylphenidate, and viloxazine in managing ADHD symptoms in children and adolescents, with medium to large effect sizes. In adults, moderate certainty evidence suggested that atomoxetine and methylphenidate offer medium efficacy in the short term for ADHD symptoms. Analyses restricted to studies with low risk of bias showed medium effect sizes of amphetamines on ADHD symptoms as rated by clinicians, with high certainty evidence. Across all age groups, the degree of concern about the safety of drug treatment varied; methylphenidate showed a favourable acceptability profile in children and adolescents, with no significant differences in tolerability compared with placebo. 

For non-drug interventions, moderate certainty evidence supported the short term efficacy of cognitive behavioural therapy in managing ADHD symptoms in adults, when rated by clinicians. Moreover, acupuncture, cognitive behavioural therapy, mindfulness, and physical training showed large effect sizes on various outcomes, although the certainty of the evidence was systematically low or very low. The low certainty ratings were primarily due to the small sample sizes used in the meta-analyses and the methodological shortcomings of the primary trials. Data on acceptability, tolerability, and side effects were generally sparse for non-drug interventions. 

For follow-up outcomes, we found no moderate or high certainty evidence for the sustained benefits of drug or non-drug interventions across age groups. Mindfulness showed some potential for long term improvements in adults, but these findings were based on limited and low certainty evidence.

### Implications for clinicians, policy makers, and other researchers 

Our study aimed to help bridge the gap between scientific evidence synthesis and practical application. To this end, we developed an interactive, open access online platform as a companion to our umbrella review, in line with the U-REACH framework.^16^ This approach ensures that findings are transparent and reproducible, and makes the data accessible and usable for people with lived experience, practitioners, and guideline developers to facilitate shared decision making for the management of ADHD symptoms.

Our results also complement current guidelines. Although we found that most non-drug interventions were supported only by low certainty evidence or small to moderate effect sizes, some of them (in particular, behavioural parent training and cognitive behavioural therapy) are often recommended in guidelines for children and adolescents with mild to moderate ADHD,^30^ or for managing associated conditions such as oppositional defiant behaviour.^31^ Other non-drug interventions (eg, acupuncture) are rarely mentioned, leaving clinicians and people with lived experience uncertain about the role of these options. Although the results of this study cannot be used to determine the value of each non-drug intervention for specific subgroups of people with ADHD, for outcomes excluded from existing meta-analyses, or in combination with drug treatment, our study can be used alongside existing guidelines to inform discussions about the risks and benefits of different treatments in shared decision making processes.

### Strengths and weaknesses of this study

Our review incorporates several methodological strengths that enhance the robustness and applicability of its findings. We did not rely on the summary statistics reported in previous meta-analyses; we instead extracted data at the randomised controlled trial level from relevant meta-analyses and re-estimated them using a uniform statistical framework. When information was unclear or missing, or when we suspected errors in the meta-analyses, we cross checked key variables against original randomised controlled trial reports, allowing us to identify and correct some inaccuracies. This also enabled us to apply stringent inclusion criteria for intervention definitions and the diagnostic status of participants. 

A limitation of this study is that our findings apply only at group level, potentially masking important individual differences in treatment response or tolerability. For example, we were unable to stratify the results by participant sex or age at the time of the ADHD diagnosis, which would have been informative for clinical practice. Future evidence synthesis using individual participant data may enable stratification of treatment effects across subgroups of individuals with ADHD. Secondly, although we sought to identify potential errors in the included meta-analyses, methodological inconsistencies between them —particularly in procedures involving subjective judgments (eg, risk of bias assessments)—may still have persisted. One onerous way to detect and better understand such inconsistencies would be to extract and compare all overlapping meta-analyses for the same combination of participants, interventions, comparators, and outcomes. Given the vast volume of available literature, this approach was not feasible. Instead, to capture the most reliable and up to date evidence, we selected the most recent and methodologically rigorous meta-analysis for each combination of participants, interventions, comparators, and outcomes. Future reviews could systematically examine the consistency of meta-analyses examining these same variables and identify the underlying factors resulting in discrepancies between meta-analyses. This examination could prioritise a subset of interventions that are the most frequently used in clinical practice—those with the highest certainty of evidence, largest efficacy, or for which there was a high risk of publication bias (ie, selective publication of studies based on the direction or significance of results). Thirdly, our review identified limited meta-analytical results for specific multimodal interventions (eg, drugs combined with psychosocial interventions) compared with a neutral control. Combination therapy is, however, common in some countries’ clinical practice and is often recommended by guidelines.^8^ Therefore, while our findings provide important insights into commonly used elements of multimodal care in clinical practice, an evidence gap remains about the combined effects of specific interventions in treatment strategies. Lastly, our review relied on access to existing, eligible meta-analyses. Consequently, our review could not capture individual randomised controlled trials on a particular intervention or outcome not yet included in a systematic review and meta-analysis, which adds to the potential incompleteness of our review, beyond the so-called file drawer effect (the tendency for studies with null or negative results to remain unpublished) that can be seen in individual meta-analyses.

The strategy for living evidence that we plan to implement could resolve several of these limitations. By regularly updating our searches, we may replace older or methodologically low quality studies with newer and more robust meta-analytical reports, and correct any calculation errors. We can also capture any previously missed meta-analyses, and progressively reduce evidence gaps as new results from reviews and trials become available, thus keeping our data accurate, current, and comprehensive. For instance, although we could only include a few meta-analyses in our review for behavioural interventions (including cognitive behavioural interventions), living individual participant data meta-analyses on this intervention type are ongoing; including such data will enrich our evidence base in the future.^32^

### Key methodological choices in relation to other studies

A key methodological choice in our umbrella review was the use of an algorithmic, rather than subjective, version of the GRADE framework, which we used to assess certainty of evidence. We did this to provide a transparent and reproducible evidence base, rather than formulating clinical recommendations, which are context dependent and require subjective judgement. Our approach is consistent with other large scale umbrella reviews^33^ and with the grading processes often used in network meta-analyses.^34^ The algorithmic GRADE system used in our review offers a standardised, context independent evaluation of evidence certainty, allowing large amounts of data from each included meta-analysis to be efficiently summarised. Moreover, despite our use of the algorithmic version of the rating system, risk of bias scoring enabled us to apply the GRADE framework to literature on drug and non-drug interventions for ADHD. Finally, by incorporating multiple indicators of potential publication bias—such as small study effects and selective outcome reporting—the GRADE framework could be applied consistently across meta-analyses, regardless of whether they included few or many primary studies. This ensured that assessments of evidence certainty were robust and comparable, even when the size and scope of the underlying meta-analyses differed.

Importantly, unlike network meta-analyses, the methodology for an umbrella review does not aim to make comparisons between different interventions.^35^ Our secondary analysis, which aimed to replicate existing network meta-analyses, showed that the network geometry of the retained network meta-analyses frequently led to substantial inconsistency between direct and indirect evidence, undermining the reliability of the results from network meta-analyses. Consequently, we decided that these findings could not be reliably interpreted and opted to focus our reporting solely on direct evidence from the network meta-analyses for our primary treatment versus control analyses. As evidence from upcoming network meta-analyses becomes available, updates of our umbrella review should enable us to analyse the comparative efficacy and safety of various interventions in a methodologically sound manner.

### Unanswered questions and future research

Because our review focused only on randomised controlled trials, these findings should be interpreted alongside data from observational studies, especially those including representative, real world participant samples, which can provide complementary insights into treatment effectiveness and generalisability.^36^ For example, within-individual design studies have shown benefits of stimulants for people with ADHD on undesirable outcomes not assessed in our review, such as rates of criminal acts or unintentional injuries.^37^ Evidence from the target trial emulation approach has also shown that use of ADHD drugs reduces the risk of death from unnatural causes,^38^ as well as lowering the risk of suicidal behaviours, substance misuse, unintentional injuries, road traffic incidents, and criminality,^39^ and slightly improves academic achievement.^40^ Currently, we are not aware of any observational evidence of this kind for non-drug interventions, but this should be pursued in future research.

Moreover, although long term use of ADHD treatments is common in clinical practice, most available randomised controlled trials are currently limited to the short term. While some discontinuation trials and long term observational studies give reassuring data on the persistent benefits and cardiovascular safety of stimulants for people with ADHD, a critical need remains for independent, pragmatic, long term randomised controlled trials that reflect real world usage, to better assess the effects of ADHD interventions over extended periods.^8^ ^41^ ^42^

### Conclusion

Our review presents the results of a comprehensive quantitative synthesis of meta-analyses of randomised evidence on effect of treatments for the management of ADHD symptoms, from preschoolers to adults. The results should inform future clinical guidelines and shared clinical decision making. We plan to periodically update and further refine the usability of the online platform with input from people with lived experience, clinicians, and other stakeholders worldwide. Furthermore, we will evaluate the impact of the platform both qualitatively and quantitatively. This will include measuring changes in stakeholder knowledge regarding ADHD treatments, as well as examining patient outcomes to determine how the platform influences real world clinical and quality of life measures.

**What is already known on this topic**

Many reviews of interventions for attention deficit/hyperactivity disorder (ADHD) exist, but the findings are often conflicting or focus on a narrow set of interventions or patient outcomes
A comprehensive and standardised review of evidence across different treatments, age groups, and outcomes has been lacking for clinicians and people with lived experience

**What this study adds**

This umbrella review indicates that although some drug interventions offer medium to large benefits in the short term for management of ADHD symptoms, they can be poorly tolerated
Some non-drug approaches had either large effect sizes but low certainty evidence, or higher evidence certainty but lower effect sizes
Findings are presented in a continuously updated online platform to make complex evidence accessible and support shared decision making in clinical practice

## Data Availability

The data generated by this work are publicly available at https://github.com/CorentinJGosling/EBI-ADHD-UR-2025. Any request regarding the dataset should be sent to cgosling@parisnanterre.fr. Additional information on the results, R codes, and raw data are publicly available at https://corentinjgosling.github.io/EBI-ADHD-UR-2025/.

## Web extra

Extra material supplied by authors

Web appendix: Supplementary files

## Notes

Contributors: CJG is the guarantor. CJG and MGA had an equal contribution. CJG, MGA, MS, RD, and SC conceived the study and drafted the manuscript. AC, CUC, DKY, HL, EO, JIS, PFP, JPAI, and JR critically reviewed the methodology. MDP, GA, AA, SA, SC, AC, PE, MD, LCF, GF, LE, APG, MH, LJ, MN, VO, VP, CP, GSDP, AT, and SJW participated in study selection and data extraction. CJG, MGA, and JR performed the data analysis. All authors critically reviewed the manuscript. The corresponding author attests that all listed authors meet authorship criteria and that no others meeting the criteria have been omitted.

Transparency: CJG affirms that the manuscript is an honest, accurate, and transparent account of the study being reported; that no important aspects of the study have been omitted; and that any discrepancies from the study as registered have been explained.

Dissemination to participants and related patient and public communities: For the dissemination of the web platform, we will rely on the extensive network of practitioners through our close contacts with the ADHD World Federation, an umbrella association of professional ADHD (attention deficit/hyperactivity disorder) organisations across the globe. We also plan to disseminate the platform to organisations of people with lived experience of ADHD, such as the Children and Adults with Attention Deficit/Hyperactivity Disorder (CHADD) in North America, Brazilian ADHD Association, and ADHD Australia, in addition to further engagement with ADHD Europe. This will ensure both efficiency and diversity in the dissemination of the evidence provided by our living umbrella review.

Provenance and peer review: Not commissioned; externally peer reviewed.
