# Supplementary material for: Benefits and harms of ADHD interventions: umbrella review and platform for shared decision making
Source: BMJ. 2025 Nov 27;391:e085875. doi: 10.1136/bmj-2025-085875 (PMC12651917; doi:10.1136/bmj-2025-085875)
Supplement: Supplementary file 1 — Web appendix: Supplementary files [file gosc085875.ww.pdf]

Supplementary Materials for "Benefits and Harms of ADHD Interventions: Umbrella Review and Platform for Shared Decision-Making".

Corentin J. Gosling, PhD, Miguel Garcia-Argibay, PhD, Michele De Prisco, MD, Gonzalo Arrondo, PhD, Anaël Ayrolles, MD, Stéphanie Antoun, MD, Serge Caparos, PhD, Ana Catalán, MD, Pierre Ellul, MD, Maja Dobrosavljevic, PhD, Luis C. Farhat, MD, Giovanna Fico, MD, Luis Eudave, MD, Annabeth P. Groenman, PhD, Mikkel Højlund, MD, Lucie Jurek, MD, Mikail Nourredine, MD, Vincenzo Oliva, MD, Valeria Parlatini, MD, Constantina Psyllou, MSc, Gonzalo Salazar-de-Pablo, MD, Anneka Tomlinson, MD, Samuel J. Westwood, PhD, Andrea Cipriani, MD, Christoph U. Correll, MD, Dong Keon Yon, MD, Henrik Larsson, PhD, Edoardo Ostinelli, MD, Jae Il Shin, MD, Paolo Fusar-Poli, MD, John P.A. Ioannidis, MD, Joaquim Radua, MD, Marco Solmi, MD, Richard Delorme, MD, Samuele Cortese, MD

S1. PRIOR checklist. **2**

https://corentinjgosling.github.io/EBI-ADHD-UR-2025/#S1_PRIOR_checklist 2

S2. Deviations from protocol. **2**

https://corentinjgosling.github.io/EBI-ADHD-UR-2025/#S2_Deviations 2

S3. Search strategies. **2**

https://corentinjgosling.github.io/EBI-ADHD-UR-2025/#S3_Search_strategies 2

S4. Selection for overlapping meta-analyses **2**

https://corentinjgosling.github.io/EBI-ADHD-UR-2025/#S4_Overlapping_meta-analyses 2

S5. Inclusion/exclusion criteria **2**

https://corentinjgosling.github.io/EBI-ADHD-UR-2025/#S5_InclExcl_criteria 2

S6. Algorithmic GRADE scoring. **2**

https://corentinjgosling.github.io/EBI-ADHD-UR-2025/#S6_GRADE_criteria 2

S7. Data analysis strategy. **3**

https://corentinjgosling.github.io/EBI-ADHD-UR-2025/#S7_Data_analytic_strategy 3

S8. List of included studies. **3**

https://corentinjgosling.github.io/EBI-ADHD-UR-2025/#S8_Included_studies 3

S9. List of excluded studies. **3**

https://corentinjgosling.github.io/EBI-ADHD-UR-2025/#S9_Excluded_studies 3

S10. Methodological quality of meta-analyses **3**

https://corentinjgosling.github.io/EBI-ADHD-UR-2025/#S10_Methodological_quality 3

S11. Primary analyses **3**

https://corentinjgosling.github.io/EBI-ADHD-UR-2025/#S11_Primary_outcomes 3

S12. Secondary analyses **3**

https://corentinjgosling.github.io/EBI-ADHD-UR-2025/#S12_Secondary_outcomes 3

S13. Long-term analyses **4**

https://corentinjgosling.github.io/EBI-ADHD-UR-2025/#S13_Longer-term 4

S14. Sensitivity: low-risk RCTs **4**

S1. PRIOR checklist

https://corentinjgosling.github.io/EBI-ADHD-UR-2025/#S1_PRIOR_checklist

S2. Deviations from protocol

https://corentinjgosling.github.io/EBI-ADHD-UR-2025/#S2_Deviations

S3. Search strategies

https://corentinjgosling.github.io/EBI-ADHD-UR-2025/#S3_Search_strategies

S4. Selection for overlapping meta-analyses

https://corentinjgosling.github.io/EBI-ADHD-UR-2025/#S4_Overlapping_meta-analyses

S5. Inclusion/exclusion criteria

https://corentinjgosling.github.io/EBI-ADHD-UR-2025/#S5_InclExcl_criteria

S6. Algorithmic GRADE scoring

https://corentinjgosling.github.io/EBI-ADHD-UR-2025/#S6_GRADE_criteria

S7. Data analysis strategy

https://corentinjgosling.github.io/EBI-ADHD-UR-2025/#S7_Data_analytic_strategy

S8. List of included studies

https://corentinjgosling.github.io/EBI-ADHD-UR-2025/#S8_Included_studies

S9. List of excluded studies

https://corentinjgosling.github.io/EBI-ADHD-UR-2025/#S9_Excluded_studies

S10. Methodological quality of meta-analyses

https://corentinjgosling.github.io/EBI-ADHD-UR-2025/#S10_Methodological_quality

S11. Primary analyses

https://corentinjgosling.github.io/EBI-ADHD-UR-2025/#S11_Primary_outcomes

S12. Secondary analyses

https://corentinjgosling.github.io/EBI-ADHD-UR-2025/#S12_Secondary_outcomes

S13. Long-term analyses

https://corentinjgosling.github.io/EBI-ADHD-UR-2025/#S13_Longer-term

S14. Sensitivity: low-risk RCTs

https://corentinjgosling.github.io/EBI-ADHD-UR-2025/#S14_Sensitivity:_Low_RoB
